# Risk Factors for Poor Prognosis of Severe Infection in Children With Idiopathic Nephrotic Syndrome: A Double-Center, Retrospective Study

**DOI:** 10.3389/fped.2021.656215

**Published:** 2021-07-14

**Authors:** Hengci Zhang, Shiyuan Qiu, Cheng Zhong, Lin Shi, Jiacheng Li, Tao Zhang, Xiaoping Zhu, Mo Wang

**Affiliations:** ^1^Department of Nephrology, Children's Hospital of Chongqing Medical University, Ministry of Education Key Laboratory of Child Development and Disorders, National Clinical Research Center for Child Health and Disorders, China International Science and Technology Cooperation Base of Child Development and Critical Disorders, Chongqing Key Laboratory of Pediatrics, Chongqing, China; ^2^Western Pediatric Development Union, Chongqing, China; ^3^Pediatric Internal Medicine Department, Chongqing You You Baobei Women's and Children's Hospital, Chongqing, China; ^4^Department of Pediatrics, The Affiliated Hospital of Guizhou Medical University, Guiyang, China

**Keywords:** idiopathic nephrotic syndrome, severe infection, risk factor, prognosis, children

## Abstract

**Background:** Infection is the most common complication of Idiopathic Nephrotic Syndrome (INS) and the main cause of INS recurrence, severe infection and even leading to mortality. The purpose of this study was to investigate the risk factors of severe infection in INS children and the clinical parameters influencing prognosis.

**Methods:** Totally 147 children with INS and concomitant infections were enrolled and classified into the severe infection group (SIG) and Non-severe infection group (Non-SIG). The clinical characteristics and auxiliary examination results were compared between the two groups, and the early-warning parameters for severe infection and risk factors for poor prognosis were evaluated.

**Results:** There were 49 patients in the SIG, 98 patients in the Non-SIG. In the SIG, the most common severe infections disease included severe pneumonia (63.6%), severe sepsis (30.6%), septic shock (4.1%). In SIG, Gram-positive bacteria (GPB) were more common, as was respiratory syncytial virus (RSV), and the three most common strains were Pseudomonas aeruginosa, Staphylococcus aureus (SA) and Staphylococcus epidermidis. There were more steroid-resistant nephrotic syndrome and combination of steroids and immunosuppressants in SIG, compared with the Non-SIG (*P* = 0.000). Patients in the SIG has lower complement 3 (C3, ≤ 0.55 g/L,) and absolute lymphocyte count (ALC, ≤ 1.5 × 10^9^/L) (*P* = 0.004). Logistic regression analysis revealed that the independent risk factors for severe infections were the combined use of immunosuppressants [95% confidence interval (CI):1.569–463.541, *P* = 0.023], steroid resistance (95% CI: 4.845–2,071.880, *P* = 0.003), C-reactive protein (CRP) ≥8 mg/L (95% CI: 43.581–959, 935.668, *P* = 0.001), and infections caused by GPB (95% CI: 27.126–2,118, 452.938, *P* = 0.002), influenza (95% CI: 2.494–1, 932.221, *P* = 0.012) and RSV (95% CI: 5.011–24 963.819, *P* = 0.007). The patients in the SIG were classified into the survival group (*N* = 39) and the mortality group (*N* = 5). Logistic regression analysis showed that white blood cell count (WBC) >15 × 10^9^/L (95% CI: 1.046–2.844, *P* = 0.033) was an independent risk factor of poor prognosis for these patients.

**Conclusions:** Resistance to steroids, combined with steroids and IS agents, and GPB infections (especially SA) are high-risk factors for severe infection in children with INS. We should monitor CRP ≥ 8 mg/L, C3 ≤ 0.55 g/L and ALC ≤ 1.5 × 10^9^/L to avoid developing severe infection. Accompanied by an increase in ANC, WBC significantly increased, suggesting a fatal infection.

## Introduction

Idiopathic nephrotic syndrome (INS) is one of the most common kidney diseases in children. It is characterized by heavy proteinuria, pitting edema, hypoalbuminemia, and hyperlipidemia. INS occurs in 1.15–16.9 per 100,000 children, and its incidence varies by ethnicity and region (1). Infection is the most common complication of INS; it hampers the treatment of the underlying kidney disease, leads to INS relapse, increases rates of unplanned hospitalizations, and even leads to increased mortality. Several recent reports have evaluated the clinical characteristics and risk factors in children with INS and infections (2, 3). However, few studies have assessed the risk factors for serious infections associated with INS and the risk factors for poor prognosis of infections. The purpose of this study was to investigate the risk factors of severe infections in children with INS and the clinical parameters leading to poor prognosis.

## Materials and Methods

### Study Population

This study enrolled children aged 0–18 years with INS and concomitant infections who were hospitalized in the Children's Hospital of Chongqing Medical University and the Affiliated Hospital of Guizhou Medical University, from January 2013 to October 2019. Written informed consent was obtained from parents or legal guardians, and this study complied with the ethical principles of the Helsinki Declaration of the World Medical Association. All the patients met the following criteria: (1) An onset age from birth to 18 years. (2) The definition of nephrotic syndrome (NS) included: ➀ nephrotic- range proteinuria: urine protein/creatinine ratio (UPCR) ≥ 200.0 (mg/mmol) in spot morning urine or 24 h urine quantification ≥50 mg/kg/day ➁ hypoproteinaemia: serum albumin <25 g/L, ➂ hyperlipidemia: serum cholesterol higher than 5.7 mmol/L and ➃ varied degrees of edema. and are necessary for diagnosis. Children were excluded from congenital nephrotic syndrome, any other secondary NS, such as Henoch–Schoenlein purpura nephritis, lupus nephritis.

### Clinical Data

The retrieved clinical data of children with INS who met the inclusion criteria were as follows: (1) Baseline characteristics, including sex, age, duration, usage, duration of steroid and immunosuppressive (IS) therapy. IS agents included cyclosporine, tacrolimus, mycophenolate, cyclophosphamide; (2) Laboratory data were collected, including complete blood count, urinalysis, alanine aminotransferase (ALT), aspartate aminotransferase (AST), alkaline phosphatase (ALP), serum urea nitrogen, serum creatinine, uric acid, serum albumin, serum potassium, serum sodium, serum chlorine, serum calcium, C-reactive protein (CRP), procalcitonin (PCT), total cholesterol (Tch), triglycerides, high-density lipoprotein (HDL), low-density lipoprotein (LDL), prothrombin time (PT), activated partial thromboplastin time (APTT), fibrinogen (Fib), D-dimer, immunoglobulin G (IgG), immunoglobulin A (IgA), immunoglobulin M (IgM), immunoglobulin E (IgE), complement 3 (C3), complement 4 (C4), and pathogens isolated by specimen culture (e.g., blood, urine, sputum, cerebrospinal fluid, and bronchoalveolar lavage fluid), antigen testing from blood, or histological material from a definite site of infection. Because complement detection helped to rule out nephropathy caused by autoimmune diseases (4), and children with INS were often accompanied by IgG deficiency, resulting in an increased risk of infection (5), almost all of the subjects in this study were tested for complement and immunoglobulin.

### Definitions

The Standard definitions of the outcome of INS were in Table 1 (6). Infection was defined as a suspected or proven infection caused by any pathogen or a clinical syndrome associated with a high probability of infection. Evidence of infection includes positive findings on clinical exam, imaging, or laboratory tests. We referred to the clinical manifestations, biochemical indicators, the definitions of severe infections in adults with INS (7, 8) and related diseases that cause INS infection in children (9–12). Finally, based on the International Guidelines for Management of Severe Sepsis and Septic Shock (2012) (13), the principle of severe infection was when children with INS have signs and symptoms of inflammation with following conditions occur: variations in general signs (at least two), variations in indicators of inflammation (at least one) and at least one of the variations in organ function, tissue perfusion or hemodynamic variations, as detailed in Table 2. The prognosis was determined based on the post-treatment conditions of the children, who were classified into good prognosis (survival group) and poor prognosis (mortality group) groups. The good prognosis was defined as symptoms and signs of inflammation are restored, without the manifestations of infection. The poor prognosis was defined as mortality.

**Table 1:** Relevant definitions in nephrotic syndrome.

	**Definition**
**Term**	
Relapse	Urine albumin 3+ or 4+ or proteinuria >40 mg/m ^2^ per h or urinary protein: creatinine ratio >2.0 (mg/mg) for 3 consecutive days
SSNS	Complete remission within 4 weeks of prednisone or prednisolone (PDN) at the standard dose (60 mg/m^2^/day or 2 mg/kg/day, maximum 60 mg/day)
SRNS	Persistent proteinuria despite 60 mg/m^2^ or 2 mg/kg for 8 weeks, after ensuring no infection or non-adherence to medication
SDNS	2 consecutive relapses occurring while weaning to alternate-day steroids or within 2 weeks of steroid discontinuation

*SSNS, steroid-sensitive nephrotic syndrome; SRNS, steroid-resistant nephrotic syndrome; SDNS, steroid-dependent nephrotic syndrome*.

**Table 2:** General variables and definitions of severe infection.

**General signs**
Fever (>38.5^°^C)
Hypothermia (<35^°^C)
Heart rate more than two SD above the normal value for age
Tachypnea (Respiratory rate higher than WHO classification for age)
Altered mental status
**Inflammatory variables**
Leukocytosis (WBC count > 12,000 μL^−1^ or higher than the normal value for age)
Leukopenia (WBC count <4,000 μL^−1^ or below the normal value for age)
Plasma C-reactive protein more than two SD above the normal value
Plasma procalcitonin more than two SD above the normal value
**Hemodynamic variables**
Arterial hypotension (SBP less than two SD below normal value for age)
**Organ dysfunction variables**
Arterial hypoxemia (PaO_2_/FiO_2_ <300)
Acute oliguria (urine output <0.5 mL kg^−1^ h^−1^ for at least 2 h despite adequate fluid resuscitation)
Coagulation abnormalities
Ileus (absent bowel sounds)
**Tissue perfusion variables**
Hyperlactatemia (Lactate above upper limits laboratory normal)
Decreased capillary refill or mottling

*SD, standard deviation; WBC, white blood cell; SBP, systolic blood pressure; WHO, World Health Organization*.

### Statistical Analysis

Statistical analysis was conducted using the SPSS 22.0 statistical software (IBM Corporation, NY, United States). In the assessment of differences in clinical parameters, categorical data were evaluated using contingency tables and the chi-square test. Quantitative data with a normal distribution were analyzed using an independent sample *t*-test. On the contrary, quantitative data that did not follow a normal distribution were analyzed using the rank-sum test. Normality testing was performed using the one-sample Kolmogorov-Smirnov test. In the binary logistic regression analysis, parameters were entered following the “forward Wald” method. The sensitivity and specificity of each parameter were compared using a univariate receiver operating characteristic (ROC) curve. For significance criteria, values of *P* < 0.05 were considered statistically significant, and values of *P* < 0.01 were considered highly statistically significant.

### Study Design

**Figure 1:**
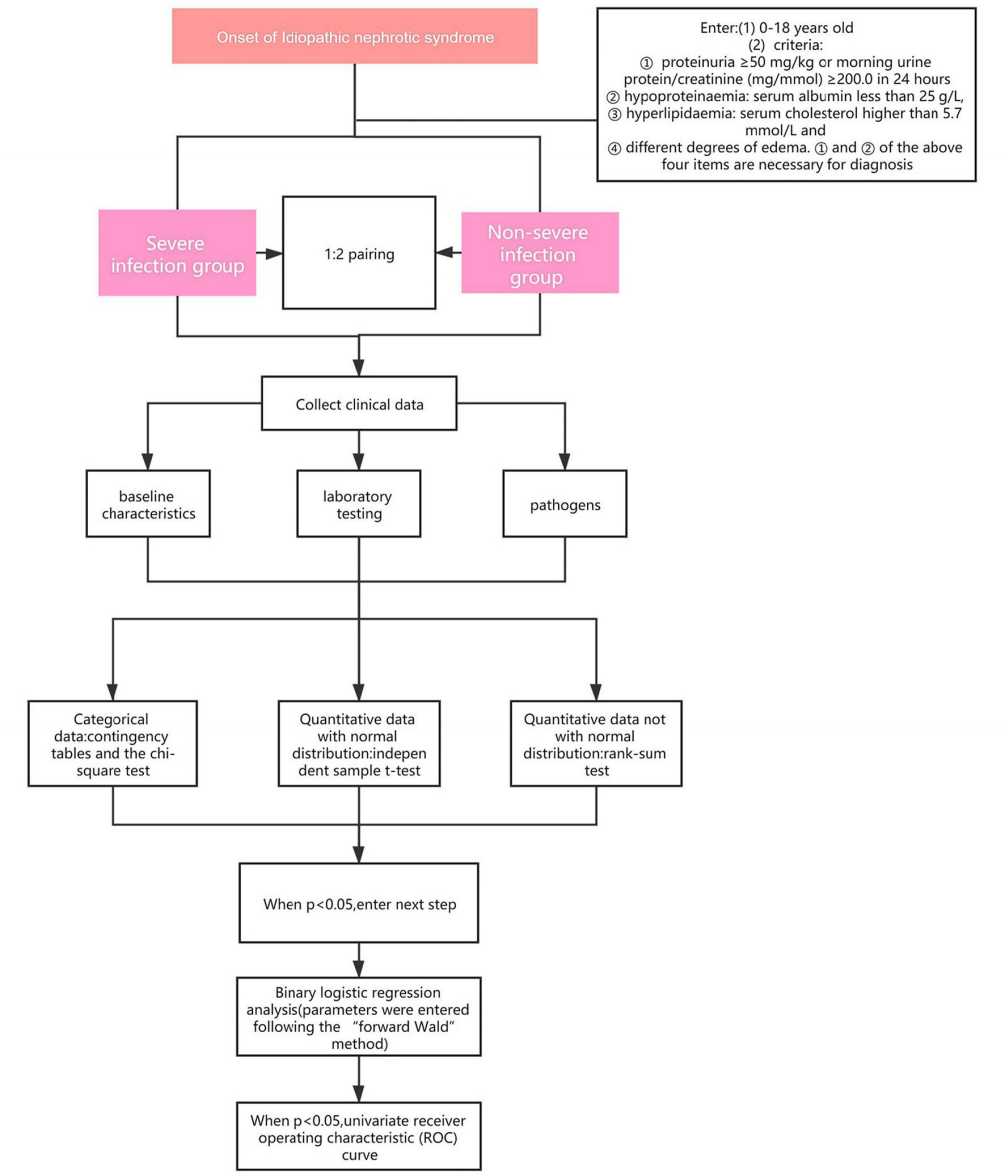
Experimental design process.

Patients who met the criteria and were complicated by infections and admitted to the Children's Hospital of Chongqing Medical University and the Affiliated Hospital of Guizhou Medical University, from January 2013 to October 2019 were into our research. According to the severity of the infections, the patients were divided into severe infection group (SIG) or Non-severe infection group (Non-SIG) (who didn't match the criteria of severe infection) and matched according to the ratio of 1:2. We retrospectively reviewed the medical data, including baseline characteristics, laboratory testing, pathogens. The flowchart of this study is shown in Figure 1.

## Results

### Baseline Characteristics

In total, 147 children, including 111 (63.8%) boys and 36 (36.2%) girls, suffering from INS with concomitant infections were enrolled in this study. The youngest child was 6 months old, and the oldest was 16.4 years old. The median age was 5.25 years old, and there was no statistically significant difference between the two groups (*P* > 0.05) (Table 3). Among the children, the shortest and longest disease durations were 0.03 and 106.13 months, respectively, and the median was 0.67 months. There were 49 children in the SIG and 98 in the Non-SIG. The SIG primarily developed severe pneumonia (*n* = 31, 63.3%), severe sepsis (*n* = 15, 30.6%), septic shock (*n* = 2, 4.1%), and severe chickenpox (*n* = 1, 2.0%); whereas, the Non-SIG primarily developed pneumonia (*n* = 81, 82.7%), urinary tract infection (UTI) (*n* = 12, 12.2%), and upper respiratory tract infection (URTI) (*n* = 5, 5.1%).

**Table 3:** Demographic characteristics, laboratory data of children with INS in the severe and non-severe infection group.

	**Non-SIG (*****N*** **=** **98)**		**SIG (*****N*** **=** **49)**		***X*^**2**^ value**	***p-*value**
	**Summary**	***N***	**Summary**	***N***		
**Baseline characteristics**						
Male	73 (74.5%)	98	38 (77.6%)	49	0.166	0.684
Female	25 (25.5%)	98	11 (22.4%)	49		
Duration (months)	0.37 (0.23, 1.78)	98	3.00 (0.67, 12.00)	49	*Z* = −3.846	0.000^**^
Age (years)	5.17 (2.52, 9.38)	98	6.00 (2.67, 10.00)	49	*Z* = −0.321	0.749
**Disease conditions**						
Initial episode	68 (69.4%)	98	18 (36.7%)	49	14.347	0.000^**^
Relapse	30 (30.6%)	98	31 (63.3%)	49		
SSNS	82 (83.7%)	98	19 (38.8%)	49	32.434	0.000^**^
SRNS	10 (10.2%)	98	25 (51.0%)	49		
SDNS	6 (6.1%)	98	5 (10.2%)	49		
**Treatments before infection**						
Duration of steroid (days)	0 (0, 0)	98	63 (0.362)	49	*Z* = −5.135	0.000^**^
MP pulse	1 (1.0%)	98	4 (8.2%)	49	3.132	0.077
**Steroid dosage per day**						
<0.5 mg/kg	82 (83.7%)	98	26 (53.1%)	49		
0.5–1.5 mg/kg	10 (10.2%)	98	15 (30.6%)	49	15.146	0.001^**^
>1.5 mg/kg	6 (6.1%)	98	8 (16.3%)	49		
IS therapy (*n*)	4 (4.1%)	98	18 (36.7%)	49	27.369	0.000^**^
Duration of IS (days)	0 (0, 0)	98	0 (0, 11)	49	−5.141	0.000^**^
**Auxiliary examinations**						
WBC (×10^9^/L)	10.18 (8.25, 12.13)	98	13.25 (9.79, 17.34)	49	*Z* = −3.850	0.000^**^
ALC (×10^9^/L)	3.27 (2.23, 4.71)	98	2.46 (1.56, 3.74)	49	*Z* = −2.889	0.004^**^
ANC (×10^9^/L)	5.46 (3.92, 8.58)	98	9.99 (6.33, 12.33)	49	*Z* = −4.442	0.000^**^
PLT (×10^9^/L)	390.0 (313.5, 459.5)	98	345.0 (244.0, 484.5)	49	*Z* = −1.448	0.147
Hb (g/L)	137 (125, 149)	98	122 (96, 144)	49	*Z* = −3.276	0.001^**^
CRP ≥ 8 mg/L (*n*)	1 (1.0%)	98	22 (44.9%)	49	47.651	0.000^**^
PCT (ng/ml)	0.05 (0.05, 0.10)	85	0.65 (0.10, 11.29)	46	*Z* = −5.812	0.000^**^
Serum albumin (g/L)	16.7 (14.7, 19.3)	98	17.2 (13.4, 22.7)	49	*Z* = −0.072	0.943
ALT (U/L)	16.0 (10.4, 23.8)	98	25.1 (14.8, 32.1)	49	*Z* = −3.493	0.000^**^
AST (U/L)	33.3 (24.7, 43.2)	98	31.2 (22.4, 51.3)	49	*Z* = −0.557	0.578
ALP (U/L)	163.6 (122.8, 199.3)	98	101.6 (77.3, 139.3)	49	*Z* = −5.519	0.000^**^
Serum uric acid (μmol/L)	326.5 (258.5, 374.0)	98	360.0 (234.0, 506.2)	49	*Z* = −2.624	0.009^**^
Serum urea nitrogen (mmol/L)	4.90 (3.78, 6.93)	98	7.64 (4.21, 12.32)	49	*Z* = −2.750	0.006^**^
Serum creatinine (μmol/L)	33.50 (25.68, 50.50)	98	45.90 (29.35, 116.25)	49	*Z* = −1.354	0.176
Serum potassium (mmol/L)	4.50 ± 0.71	98	4.04 ± 0.72	49	*t* = 3.670	0.000^**^
Serum sodium (mmol/L)	136.30 (132.55, 138.75)	98	132.20 (128.95, 137.10)	49	*Z* = −3.048	0.002^**^
Serum chlorine (mmol/L)	103.85 (100.20, 106.83)	98	101.50 (96.50, 105.05)	49	*Z* = −2.554	0.011^*^
Serum calcium (mmol/L)	1.94 (1.82, 2.05)	98	1.87 (1.73, 2.09)	49	*Z* = −1.459	0.145
Tch (mmol/L)	10.76 ± 3.10	98	8.86 ± 3.53	49	*t* = 3.337	0.001^**^
Triglyceride (mmol/L)	2.98 (2.01, 4.36)	98	3.14 (2.43, 4.17)	49	*Z* = −0.489	0.625
LDL (mmol/L)	7.86 ± 2.96	98	5.66 ± 3.23	49	*t* = 4.112	0.000^**^
HDL (mmol/L)	1.71 (1.23, 2.21)	98	1.50 (0.81, 2.16)	49	*Z* = −1.724	0.085
IgG (g/L)	2.24 (1.36, 3.02)	97	1.88 (0.83, 4.29)	48	*Z* = −0.534	0.594
IgA (g/L)	0.85 (0.63, 1.35)	97	0.89 (0.62, 1.30)	48	*Z* = −0.089	0.929
IgM (g/L)	1.76 (1.31, 2.40)	97	1.49 (1.09, 2.22)	48	*Z* = −1.589	0.112
IgE (g/L)	176.0 (46.2, 458.5)	97	76.3 (23.8, 372.0)	48	*Z* = −1.790	0.073
C3 (g/L)	0.99 ± 0.21	97	0.85 ± 0.30	48	*t* = 2.947	0.004^**^
C4 (g/L)	0.24 (0.19, 0.30)	97	0.23 (0.17, 0.27)	48	*Z* = −0.854	0.393
PT (s)	10.4 (9.7, 11.1)	87	11.2 (10.2, 13.3)	48	*Z* = −3.151	0.002^**^
APTT (s)	31.00 (26.90, 35.70)	87	36.75 (28.15, 53.23)	48	*Z* = −2.882	0.004^**^
Fib (g/L)	5.97 ± 1.70	87	5.31 ± 2.02	48	*t* = 2.023	0.045^*^
D-dimer	1.26 (0.58, 2.31)	87	1.83 (0.69, 5.80)	48	*Z* = −1.836	0.066

* *p < 0.05*,

** *p < 0.01*.

*Continuous parameters were expressed as (X̄ ± s) or P50 (P25, P75), and discrete parameters were expressed as a percentage (%). SIG, severe infection group; MP, methylprednisolone; IS, immunosuppressive; WBC, white blood cells; ALC, absolute lymphocyte count; ANC, absolute neutrophil count; PLT, platelets count; Hb, hemoglobin; CRP, C-reactive protein; PCT, procalcitonin; ALT, alanine aminotransferase; AST, aspartate aminotransferase; ALP, alkaline phosphatase; Tch, total cholesterol; LDL, low-density lipoprotein; HDL, high-density lipoprotein; PT, prothrombin time; APTT, activated partial thromboplastin time; Fib, fibrinogen*.

### Clinical Data Analysis

Through statistical analysis of clinical data, we found that there were many differences between SIG and Non-SIG. The percentage of relapsed cases and SRNS in SIG were higher than those Non- SIG (63.3 vs. 30.6%, 51.0 vs. 10.2%, respectively). The number of steroids (≥0.5 mg/kg) users and immunosuppressant users in SIG was significantly higher than that in the Non-SIG (46.9 vs. 16.3%, 36.7 vs. 4.1%). There were 18 cases using immunosuppressive agents, including 12 cases using tacrolimus, of which 2 cases were combined with mycophenolate mofetil and 1 case was combined with cyclophosphamide. The remaining 4 cases used mycophenolate mofetil alone and 2 cases used cyclophosphamide alone. Steroids and immunosuppressants for SIG were used longer than for Non -SIG. In the auxiliary examination, we also found that there were significant differences between the two groups. The ANC, PCT, PT, and APTT in the SIG were significantly higher than those in the Non-SIG, while ALC, Tch, LDL, and C3 were significantly lower than those in the Non-SIG. Other indicators were also significantly different, such as WBC, CRP, uric acid, urea nitrogen, and other indicators SIG are higher than Non-SIG. The SIG group's Fib, serum potassium, sodium, etc. were lower than the Non-SIG group (Table 3).

### Analysis of Pathogens and Drug Resistance in the Non-SIG and SIG

Based on the data from blood, sputum, and urine cultures obtained from 147 children with INS and infections during hospitalization, 30 of them were detected and 46 strains were isolated. The three most common types in the SIG were Pseudomonas aeruginosa (three strains), Staphylococcus aureus (SA) (three strains), and Staphylococcus epidermidis (three strains). On the contrary, the three most common types in the Non-SIG were Haemophilus influenzae (three strains), Streptococcus pneumonia (SP) (three strains), and Enterococcus faecium (three strains). The detection rate of Gram-positive bacteria (GPB) was higher in the SIG than in the Non-SIG (Table 4). The pathogen profiles indicated that the SIG was more likely to be infected with GPB, respiratory syncytial virus (RSV) and influenza viruses (FLu), and fungi than the Non-SIG (*P* < 0.05). In total, 10 drug-resistant strains were detected in the two groups. The incidence of Methicillin-resistant strains was significantly higher in the SIG (*n* = 4) than in the Non-SIG (*n* = 0) (*P* = 0.000).

**Table 4:** Pathogen spectrum and the analysis in the severe and non-severe infection group.

**Pathogens**	**Non-severe infection group**	**Severe infection group**	**Total (*N*, %)**	**X^**2**^ value**	***p-*value**
Gram-negative bacteria	12	14	26 (56.52)	2.577	0.108
*Morganella*	1	2	3 (6.52)		
*Escherichia coli*	3	1	4 (8.70)		
*Acinetobacter baumannii*	1	2	3 (6.52)		
*Enterobacter cloacae*	0	1	1 (2.17)		
*Klebsiella pneumonia*	0	2	2 (4.35)		
*Pseudomonas aeruginosa*	1	3	4 (8.70)		
*Haemophilus influenzae*	3	2	5 (10.87)		
*Haemophilus parainfluenzae*	2	0	2 (4.35)		
*Elizabethkingia*	0	1	1 (2.17)		
*Pantoea agglomerans*	1	0	1 (2.17)		
Gram-positive bacteria	7	13	20 (43.48)	10.256	0.001
*Staphylococcus aureus*	1	3	4 (8.70)		
*Staphylococcus epidermidis*	0	3	3 (6.52)		
*Staphylococcus hominis*	0	2	2 (4.35)		
*Staphylococcus haemolyticus*	0	1	1 (2.17)		
*Streptococcus pneumoniae*	3	2	5 (10.87)		
*Enterococcus faecium*	3	2	5 (10.87)		
Fungus	0	4	4	5.429	0.020
Virus	18	25	43		
*Cytomegalovirus*	8	5	13 (30.23)	0.011	0.918
*Epstein-Barr virus*	2	2	4 (9.30)	0.032	0.858
*Adenovirus*	1	0	1 (2.33)	0.000	1.000
*Influenza virus*	3	7	10 (23.26)	4.842	0.028
*Parainfluenza virus*	3	3	6 (13.95)	0.195	0.658
*Respiratory syncytial virus*	1	8	9 (20.93)	10.785	0.001
Mycoplasma pneumoniae	7	1	8	0.8100	0.368

### Risk Factor Analysis for Severe Infections in INS

To identify the risk factors of severe infections in children with INS, the aforementioned clinical parameters that were statistically different between the two groups were entered into the binary logistic regression analysis model. The results obtained using the “forward Wald” method were shown in Table 5. Binary logistic regression analysis showed that the combined use of immunosuppressants (OR: 26.969, 95% CI: 1.569–463.541), CRP ≥8 mg/L (OR: 6,468.029, 95% CI: 43.581–959,935.668), steroid resistance (OR: 100.191, 95% CI: 4.845–2,071.880), infection with gram-positive cocci (OR: 7,580.545, 95% CI: 27.126–2,118,452.938), infection with influenza virus (OR: 69.426, 95% CI: 2.494–1,932.221), and infection with respiratory syncytial virus (OR: 353.681, 95% CI: 5.011–24,963.819) were independent risk factors for severe infections in children with INS. ALC (increase > 1.5 × 10^9^/L) (OR: 0.356, 95% CI: 0.188–0.675) and complement C3 (increase > 0.55 g/L) (OR: 0.000, 95% CI: 0.000–0.276) were protective factors.

**Table 5:** Binary logistic regression analysis of risk factors of severe infections in children with INS.

	**Coefficient (B)**	**Wald value**	***p***	**OR**	**95% Confidence interval for EXP (B)**	
					**Lower limit**	**Upper limit**
Steroid Combined with IS	3.295	5.155	0.023	26.969	1.569	463.541
SSNS		9.375	0.009			
SRNS	4.607	8.886	0.003	100.191	4.845	2,071.880
SDNS	1.979	1.208	0.272	7.233	0.212	246.345
Increased ALC	−1.033	10.025	0.002	0.356	0.188	0.675
CRP ≥ 8 mg/L	8.775	11.831	0.001	6,468.029	43.581	959,935.668
Increased C3	−8.205	5.405	0.020	0.000	0.000	0.276
Infection with gram-positive bacteria	8.933	9.662	0.002	7,580.545	27.126	2,118,452.938
Infection with influenza virus	4.240	6.243	0.012	69.426	2.494	1,932.221
Infection with RSV	5.868	7.301	0.007	353.681	5.011	24,963.819
Constant	3.318	1.269	0.260	27.612		

*IS, Immunosuppressant; ALC, absolute lymphocyte count; RSV, respiratory syncytial virus*.

**Figure 2:**
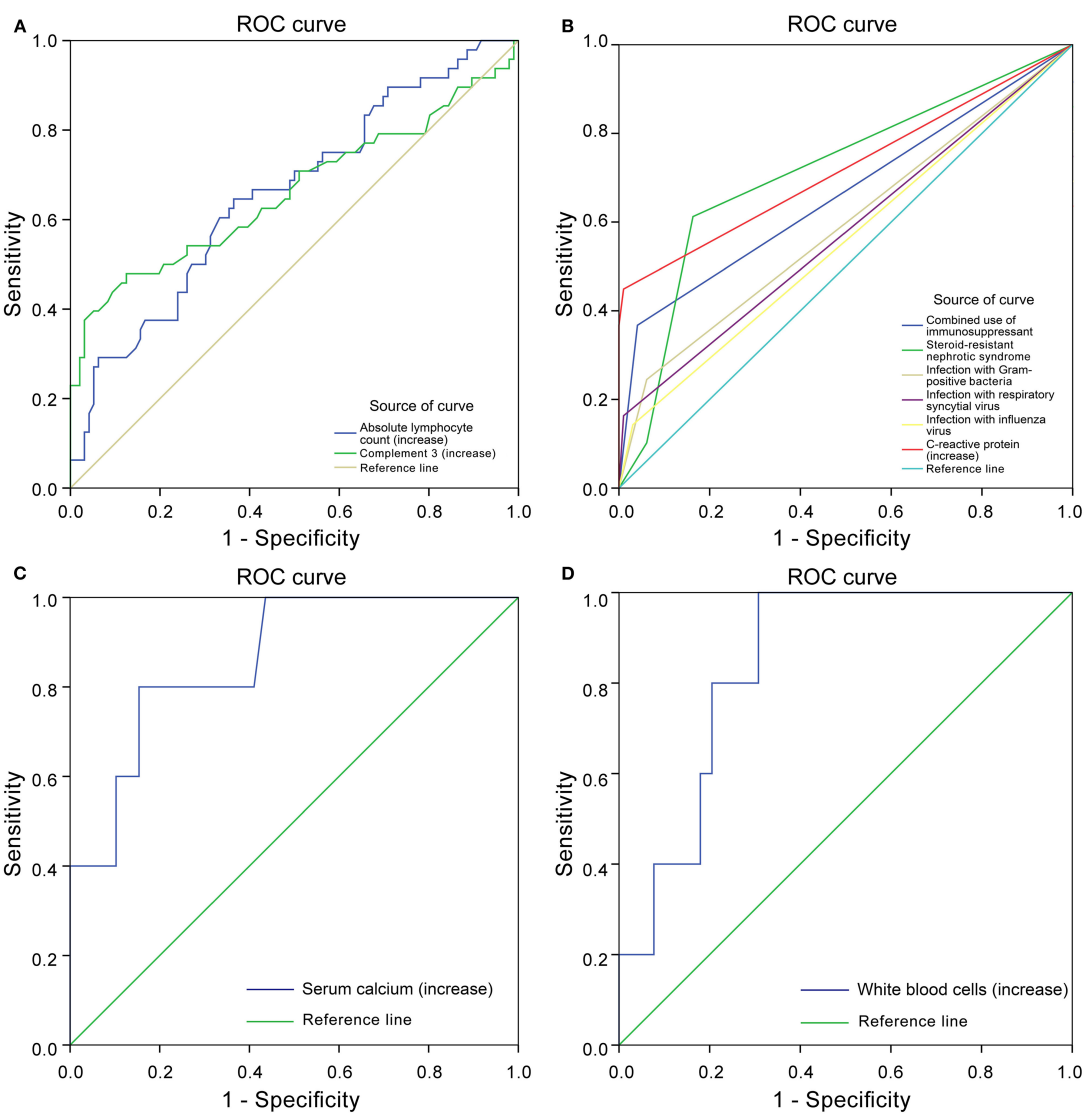
Receiver operating characteristics (ROC) curve. Protective factors of severe infection **(A)**. Risk factors of severe infection **(B)**. Protective factors for poor prognosis of severe infection **(C)**. Risk factors for poor prognosis of severe infection **(D)**.

Through the ROC curve model analysis, it was found that when children with nephropathy were co-infected, the risk of infection progression often needed to consider the combined effect of multiple influencing factors at the same time. The sensitivity and specificity reflected by the area under the ROC curve (AUC) showed that when infection occurred in children with INS, when one or more factors including steroid resistance, IS treatment and increased CRP were combined, a more severe infection was very likely to occur (Figures 2A,B).

### Analysis of Prognosis of INS Patients With Concomitant Severe Infections

In the SIG, 39 patients survived, five died, and five gave up treatment. Five patients who gave up treatment were excluded because of incomplete data and unclear prognosis. The causes of death included multiple organ failure syndromes (2 cases), brain herniation (1 case), and circulatory failure (2 cases). The patients were classified into the survival group (*N* = 39) and the mortality group (*N* = 5). The statistically significant parameters between two groups by univariate analysis entered into the binary logistic regression analysis model. There was no significant difference in baseline characteristics, treatment, and disease condition between the two groups. However, in the auxiliary examination, WBC, Serum chlorine, and ANC in the mortality group were significantly higher than those in the survival group, and ALT, AST, serum calcium, IgG, IgA, and C3 were significantly lower than those in the survival group (Table 6). Binary logistic regression analysis showed that WBC (increased > 15 × 10^9^/L) (OR: 1.725, 95% CI: 1.046–2.844) is an independent risk factor for the poor prognosis of INS children with severe infections, often accompanied by an increase in the ANC(>10 × 10^9^/L), whereas serum calcium level (increase > 1.30 mmol/L) (OR: 0.000, 95% CI: 0.000–0.698) was a protective factor. The area under the ROC curve shows that serum calcium and WBC are highly specific and sensitive in predicting the risk of poor prognosis (Figures 2C,D).

**Table 6:** Univariate analysis of risk factors for poor prognosis of SIG in children with INS.

	**Survival group (*****N*** **=** **39)**		**Mortality group (*****N*** **=** **5)**		**X^**2**^ value**	***p-*value**
	**Summary**	***N***	**Summary**	***N***		
WBC (×10^9^/L)	12.75 (8.98, 15.99)	39	21.20 (15.78, 30.96)	5	*Z* = −2.496	0.013^*^
PLT (×10^9^/L)	366 (298, 499)	39	155 (91, 461)	5	*Z* = −1.498	0.134
Hb (g/L)	126 (106, 145)	39	80 (68, 125)	5	*Z* = −1.998	0.046
ALC (×10^9^/L)	2.36 (1.57, 3.43)	39	2.47 (1.38, 4.43)	5	*Z* = −0.296	0.767
ANC (×10^9^/L)	9.27 (5.18, 11.39)	39	12.21 (10.84, 20.15)	5	*Z* = −2.034	0.042^*^
CRP ≥ 8 mg/L (*n*)	17 (43.6%)	39	4 (80.0%)	5		0.176^Δ^
PCT (ng/ml)	0.43 (0.10, 8.65)	39	5.52 (0.62, 45.64)	5	*Z* = −1.423	0.155
Serum albumin (g/L)	17.06 (14.30, 23.80)	39	12.60 (10.20, 15.35)	5	*Z* = −1.029	0.303
ALT (U/L)	25.10 (15.10, 28.50)	39	13.40 (9.75, 67.75)	5	*Z* = −2.663	0.008^**^
AST (U/L)	31.20 (25.40, 46.50)	39	22.10 (18.65, 242.05)	5	*Z* = −2.422	0.015^*^
ALP (U/L)	102.60 (81.20, 137.50)	39	78.00 (63.05, 164.50)	5	*Z* = −1.091	0.275
Serum uric acid (μmol/L)	346 (224, 485)	39	360 (271, 449)	5	*Z* = −0.185	0.853
Serum urea nitrogen (mmol/L)	6.33 (3.70, 11.70)	39	8.20 (5.67, 28.08)	5	*Z* = −1.183	0.237
Serum creatinine (μmol/L)	40.60 (27.00, 74.50)	39	82.00 (37.00, 284.75)	5	*Z* = −1.738	0.082
Serum potassium (mmol/L)	4.11 ± 0.68	39	3.61 ± 1.04	5	*t* = 1.440	0.157
Serum sodium (mmol/L)	131.5 (129.2, 136.7)	39	132.4 (122.0, 141.0)	5	*Z* = −0.129	0.897
Serum chlorine (mmol/L)	101.9 (96.0, 104.1)	39	109.6 (101.1, 114.6)	5	*Z* = −2.238	0.025^*^
Serum calcium (mmol/L)	1.91 (1.75, 2.09)	39	1.67 (1.29, 1.78)	5	*Z* = −2.626	0.009^**^
Tch (mmol/L)	9.24 ± 3.62	39	8.36 ± 2.69	5	*t* = 0.519	0.607
Triglyceride (mmol/L)	3.10 (2.38, 3.86)	39	3.73 (3.05, 10.27)	5	*Z* = −1.110	0.267
LDL (mmol/L)	5.89 ± 3.30	39	5.05 ± 3.10	5	*t* = 0.541	0.591
HDL (mmol/L)	1.53 (0.94, 2.04)	39	0.81 (0.56, 1.33)	5	*Z* = −1.923	0.054
IgG (g/L)	2.06 (0.96, 4.02)	39	0.66 (0.51, 0.86)	5	*Z* = −2.644	0.008^**^
IgA (g/L)	0.91 (0.66, 1.31)	39	0.48 (0.40, 0.62)	5	*Z* = −2.570	0.010^*^
IgM (g/L)	1.48 (1.08, 2.51)	39	1.74 (0.82, 1.85)	5	*Z* = −0.481	0.631
IgE (g/L)	81.30 (21.30, 410.00)	39	32.20 (27.66, 187.95)	5	*Z* = −0.795	0.427
C3 (g/L)	0.88 ± 0.30	39	0.63 ± 0.09	5	*t* = 3.940	0.001^**^
C4 (g/L)	0.22 (0.16, 0.27)	39	0.25 (0.23, 0.26)	5	*Z* = −0.981	0.326
PT (s)	11.05 (10.18, 12.83)	39	11.20 (10.00, 13.20)	5	*Z* = −0.114	0.909
APTT (s)	36.30 (26.78, 53.83)	39	37.50 (32.40, 44.20)	5	*Z* = −0.265	0.791
Fib (g/L)	5.56 ± 1.97	39	5.22 ± 1.99	5	*t* = 0.369	0.714
D-dimer	1.44 (0.55, 3.20)	39	3.10 (0.86, 32.25)	5	*Z* = −1.137	0.256

* *p < 0.05*,

** *p < 0.01*.

Δ *Fisher exact method*.

*Continuous parameters were expressed as (X̄ ± s) or P50 (P25, P75), and discrete parameters were expressed as a percentage (%). SIG, severe infection group; WBC, white blood cells; ALC, absolute lymphocyte count; ANC, absolute neutrophil count; Hb, hemoglobin; PLT, platelets count; CRP, C-reactive protein; PCT, procalcitonin; ALT, alanine aminotransferase; AST, aspartate aminotransferase; ALP, alkaline phosphatase; Tch, total cholesterol; LDL, low-density lipoprotein; HDL, high-density lipoprotein; PT, prothrombin time; APTT, activated partial thromboplastin time; Fib, fibrinogen*.

## Discussion

Infection is one of the main complications of childhood idiopathic nephrotic syndrome, which can lead to frequent relapses of INS, treatment failures, and even death (14). Children with INS are more likely to have a poor prognosis after infection due to the primary disease and therapeutic drugs. At present, most studies on children's INS focus on the analysis of risk factors for infection, and there is a lack of large-scale reports on the risk factors and outcome factors for severe infection. Referring to the definition of severe infection in adult INS and the indicators of severe infection in children (7, 8), we defined the manifestations of severe infection in children with INS and analyzed the relevant risk factors.

In this study, it was observed that pneumonia and urinary tract infection were dominant in Non-severely infective INS children, while severe pneumonia, sepsis was dominant in severely infective INS children. In Taiwan (15), pneumonia is the main type of infection in children with INS, which is consistent with the Non-SIG in our study, while in India, Saudi Arabia, and other regions (2, 16, 17), the upper respiratory tract infection is the main type, but these studies did not grade the infection. Although in Israel (18), the infections were mainly pneumonia and bacteremia/sepsis, but they defined the infection as a serious bacterial infection, which has limitations. From our research, it is found that both severe and Non-severe infections are mainly respiratory infections. Therefore, our treatment should be more active when pneumonia is complicated. We found that the pathogen spectrum of the two groups is significantly different, and GPB are an independent risk factor for severe infections. In SIG, the respiratory pathogens are mainly SA, while Non-SIG is mainly composed of SP and Haemophilus influenzae, and it is more common for SIG strains to have drug resistance. Therefore, when patients are infected with methicillin-resistant SA or other methicillin-resistant GPB, they are more likely to cause serious infections. So, children with INS must vaccinate pneumococcal vaccine. Previous studies have confirmed that the use of high cumulative doses of steroids and IS agents (8, 17, 19–23) all increase the risk of INS infection. Besides, we found that children with SRNS or relapse are more susceptible to severe infection, and the risk of severe infection is higher when treated with steroids combined with immunosuppressive agents such as tacrolimus or mycophenolate mofetil. From the auxiliary inspection point of view, SIG has higher CRP and PCT, but their Tch and LDL are lower. The Tch and LDL of the two groups were higher than the normal range, but studies have shown that inflammation can cause a decrease in Tch (24), so the LDL of SIG is lower than that of Non-SIG. When the Tch and LDL of infected children with INS decrease, we need to be vigilant. SIG's PT and APTT are also higher, suggesting that when there is a problem with the coagulation function of Non-SIG, we need to be alert to the progress of the infection, and even cause diffuse intravascular coagulation. C3 (<0.55 g/L) and ALC (<1.5 × 10^9^/L) are predictors of severe infections, which had not been previously reported. We believe that this is because the use of high-dose steroids and IS agents and INS itself will cause ALC and C3 to decrease. Co-infection will further reduce the ALC and C3 (25–27). Therefore, When C3 ≤ 0.55 g/L or ALC ≤ 1.5 × 10^9^/L, the infection should be timely and controlled to avoid further aggravation.

Regarding prognosis, we found that children in the mortality group had lower immunity. The serum calcium of the mortality group was significantly reduced, which is one of the factors of poor prognosis. In INS, hypoalbuminemia is often accompanied by a decrease in serum calcium. The cause of hypocalcemia may not only be caused by INS itself, but also infection. Serum calcium lower than 1.30 mmol/L may cause death. WBC (>15 × 10^9^/L) was the only independent risk factor for poor prognosis, no similar results have been reported in children with INS, whereas reports involving adults with INS have shown that hypotension and decreased platelet count were risk factors for a poor prognosis (14). Although decreased ALC is a risk factor for severe infection, however, due to an increase in ANC, the resulting increase in white blood cells became an early warning indicator of fatal severe infection, especially the increase in WBC increased (>15 × 10^9^/L).

Several limitations of this study should be mentioned. First, although we have found some risk factors of severe infection in children with INS, as a retrospective study, it might lead to the bias in data collection and uniform availability of information from the medical records. Second, the age span of children in this study was large, and the results could not well-reflect the clinical characteristics of children with INS complicated with severe infection in each age period. We tried to divide the children into infant, childhood and adolescence group based on tanner staging. However, due to the relatively small sample size, it was hard to perform statistical analysis. In the future, we will expand the sample size and design a prospective study based on the results of this study, so as to avoid poor prognosis of children with INS caused by severe infection, and to find more risk factors of severe infection in children with INS in different age period.

In summary, our double-center retrospective study showed that resistance to steroids, combined use of steroids and IS agents, and GPB infections (especially SA) are high risk factors for severe infection. We should monitor CRP ≥ 8 mg/L, C3 ≤ 0.55 g/L and ALC ≤ 1.5 × 10^9^/L to avoid developing severe infection. The WBC of severely infected patients increases significantly, especially when ANC increases, which can lead to fatal infections.

## Data Availability Statement

The raw data supporting the conclusions of this article will be made available by the authors, without undue reservation.

## Ethics Statement

The studies involving human participants were reviewed and approved by Ethics Committee of the Children's Hospital of Chongqing Medical University (reference number 2020-12, 2020-03-24). Written informed consent to participate in this study was provided by the participants' legal guardian/next of kin.

## Author Contributions

MW and XZ designed this study. HZ and SQ wrote the draft. CZ, LS, and TZ has participated in data collection and case follow-up. JL designed the statistical analysis of the study. All authors contributed to the article and approved the submitted version.

## Conflict of Interest

The authors declare that the research was conducted in the absence of any commercial or financial relationships that could be construed as a potential conflict of interest.
